# Posterior axis formation requires *Dlx5/Dlx6* expression at the neural plate border

**DOI:** 10.1371/journal.pone.0214063

**Published:** 2019-03-19

**Authors:** Nicolas Narboux-Neme, Marc Ekker, Giovanni Levi, Eglantine Heude

**Affiliations:** 1 Département Adaptations du Vivant, Centre National de la Recherche Scientifique UMR 7221, Muséum National d’Histoire Naturelle, Paris, France; 2 Department of Biology, Centre for Advanced Research in Environmental Genomics, University of Ottawa, Ottawa, Ontario, Canada; Laboratoire de Biologie du Développement de Villefranche-sur-Mer, FRANCE

## Abstract

Neural tube defects (NTDs), one of the most common birth defects in human, present a multifactorial etiology with a poorly defined genetic component. The *Dlx5* and *Dlx6* bigenic cluster encodes two evolutionary conserved homeodomain transcription factors, which are necessary for proper vertebrate development. It has been shown that *Dlx5/6* genes are essential for anterior neural tube closure, however their role in the formation of the posterior structures has never been described. Here, we show that *Dlx5/6* expression is required during vertebrate posterior axis formation. *Dlx5* presents a similar expression pattern in neural plate border cells during posterior neurulation of zebrafish and mouse. *Dlx5/6*-inactivation in the mouse results in a phenotype reminiscent of NTDs characterized by open thoracic and lumbar vertebral arches and failure of epaxial muscle formation at the dorsal midline. The *dlx5a/6a* zebrafish morphants present posterior NTDs associated with abnormal delamination of neural crest cells showing altered expression of cell adhesion molecules and defects of motoneuronal development. Our findings provide new molecular leads to decipher the mechanisms of vertebrate posterior neurulation and might help to gather a better understanding of human congenital NTDs etiology.

## Introduction

Neural tube defects (NTDs) correspond to a wide spectrum of common congenital disorders resulting from total or partial failure of neural tube closure during early embryogenesis. NTDs affect from 0.3 to 200 per 10 000 births worldwide [1] and vary in type and severity depending on the neural tube levels affected along the antero-posterior axis. Anterior and posterior neural tube defects lead respectively to brain (ie. exencephaly, anencephaly) or spinal cord malformations (ie. spina bifida); complete antero-posterior defect in neural tube closure is at the origin of a more severe form of NTD termed craniorachischisis (reviewed in [2, 3]). The origins of NTDs have been associated to genetic and/or environmental factors and more than 200 mutant mice have been reported to present different forms of neural tube malformations [4, 5]. However, given the complexity of the NTD spectrum, there has been limited progress in defining the molecular basis of these conditions.

In vertebrates, neural tube defects originate from a failure in morphogenetic events taking place during the neurulation process. In mammalian embryos, neurulation involves two distinct morphogenetic processes along the rostro-caudal axis, known as primary and secondary neurulations. Primary neurulation refers to neural tube formation originating from folding of an open neural plate that forms the central lumen in the anterior part of the embryo. In contrast, secondary neurulation is characterized by mesenchymal condensation and cavitation in the posterior axis caudal to the tail bud [6, 7]. In zebrafish, neurulation occurs homogeneously along the rostro-caudal axis by epithelial condensation forming the neural plate, followed by cavitation as observed during secondary neurulation in mammals [8, 9]. Zebrafish neurulation has been linked either to primary or to secondary neurulation of higher vertebrates [6, 7]. However, the morphogenetic similarities observed between neurulation in teleosts and other vertebrates indicate that zebrafish neural tube formation rather correspond to primary neurulation and constitutes a viable model to study vertebrate neural tube development [6, 10].

The general primary neurulation dynamic seems conserved among vertebrates and is characterized by convergent movement of the neural plate borders (NPB) toward the dorsal midline to generate the neural tube with a central lumen [6, 9]. NPB cells constitute a competence domain, established between neural and non-neural ectoderm, that delineates the presumptive domain at the origin of migratory neural crest cells (NCCs) and responsible for neural tube closure [11, 12].

*Dlx* genes, the vertebrate homologues of *distal-less (dll)* in arthropods, code for an evolutionary conserved group of homeodomain transcription factors. The mouse and human *Dlx* gene system is constituted by three closely associated bigenic clusters located on the same chromosomes as *Hox* genes clusters. In teleost, the *dlx* clusters are arranged on chromosomes similarly to their tetrapod *Dlx* counterparts [13]. The most probable scenario suggests that *Dlx* genes have arisen from an ancestral *dll* gene as a result of gene duplication events [14]. Data indicate that *Dlx* genes from a same cluster, such as *Dlx5* and *Dlx6* paralogs, present redundant functions during vertebrate development [15–18].

It has been shown that *Dlx5* is one of the earliest NPB markers defining the limit of the neural plate during neurulation of mouse, chick, frog and zebrafish [18–24]. Inactivation of *Dlx5* in mouse results in a frequent exencephalic phenotype suggesting defects of anterior neural tube closure [16], however the mice do not present obvious posterior axis malformations. As *Dlx5* and *Dlx6* have partially redundant functions, it has been necessary to simultaneously inactivate both genes to fully reveal their roles during development. Functional analyses of *Dlx5/Dlx6* inactivation in mice, avian and fish have demonstrated evolutionary conserved roles in appendage morphogenesis, in neurogenesis, in the development of the face and of the reproductive system [16–18, 25–31]. *Dlx5/6*^-/-^ mice also present midline-fusion abnormalities including hypospadias, failure of anterior neuropore closure and tail malformations [17, 31, 32]. However, the origin of the latter phenotype has never been described.

Here we show that simultaneous inactivation of *Dlx5* and *Dlx6* in zebrafish and mouse results in posterior NTDs. Our data indicate a conserved role for *Dlx5/6* in posterior neurulation in vertebrates and suggest that genetic pathways involving these genes might be implicated in syndromic forms of human midline defects.

## Materials and methods

### Ethical statement

All experiments with zebrafish were performed according to the guidelines of the Canadian Council on Animal Care and were approved by the University of Ottawa animal care committee (institutional licence #BL 235 to ME). All efforts were made to minimize suffering; manipulations on animals were performed with the anaesthetic drug tricaine mesylate (ethyl 3-aminobenzoate methanesulfonate; Sigma-Aldrich, Oakville, ON, Canada). Embryos were killed with an overdose of the latter drug.

Procedures involving mice were conducted in accordance with the directives of the European Community (council directive 86/609) and reviewed and approved by the “Cuvier” ethical committee of the Muséum National d’Histoire Naturelle and the French Ministry of Agriculture (council directive 87–848, 19 October 1987, permissions 00782 to GL).

### Animal maintenance

Zebrafish and their embryos were maintained at 28.5°C according to methods described in [33]. Wild-type adult zebrafish were kept and bred in circulating fish water at 28.5°C with a controlled 14-h light cycle. Wild type (WT), controls and morphant embryos were raised at similar densities in embryo medium in a 28.5°C incubator. Embryos were treated with 0.0015% 1-phenyl 2-thiourea (PTU) to inhibit melanogenesis and were killed with an overdose of tricaine mesylate for analysis.

Mice were housed in light, temperature (21°C) and humidity controlled conditions; food and water were available ad libitum. WT animals were from Charles River France. The mouse heterozygous strain *Dlx5/6*^*+/-*^ was maintained on a hybrid genetic background resulting from crosses between C57BL/6N females and DBA/2JRj males (B6D2N; Janvier Labs, France); the mice are viable and present a normal phenotype [25, 27, 28, 32]. The *Dlx5/6*^*-/-*^ homozygous foetuses were obtained by crossing *Dlx5/6*^*+/-*^ mice and our controls have been either WT or *Dlx5/6*^*+/-*^ littermates.

### Morpholino-mediated knockdown

The morpholino-mediated knockdown was validated as previously described [18]. We performed injection or co-injection of *dlx5a* and/or *dlx6a* MOs in 1 cell-stage embryos (0.4 mM or 0.8 mM). The 5’-untranslated region of *dlx* genes has been used to design translation-blocking antisense MOs against *dlx* transcripts. The following translation-blocking MOs were obtained from Gene Tools (LLC, Philomath, OR, USA): *dlx5a* MO 5’-TCCTTCTGTCGAATACTCCAGTCAT-3’; *dlx6a* MO 5’-TGGTCATCATCAAATTTTCTGCTTT-3’.

Splice-blocking MOs targeting exon 2 excision were also designed to confirm the posterior axis phenotype obtained using the translation-blocking MOs: *dlx5a* e2i2 MO 5’-TATTCCAGGAAATTGTGCGAACCTG-3’; *dlx6a* e2i2 MO 5’-AAATGAGTTCACATCTCACCTGCGT-3’ (from Gene Tools, LLC).

As controls, we injected water or 1.6 mM of control standard MO (Gene Tools) that targets a human beta-globin intron mutation that causes beta-thalessemia. (Gene Tools 5’-CCTCTTACCTCAGTTACAATTTATA-3’). To ensure MOs specificity, rescue of morphant phenotypes was performed by co-injecting the corresponding *dlx5a/dlx6a* transcripts mutated on the MO target site (*dlx5a* MO binding site T(ATG)ACTGGAGTATTCGACAGAAGGA, *mutdlx5a* sequence C(ATG)ACGGGTGTTTTTGATAGGAGGA; *dlx6a* MO binding site AAAGCAGAAAATTTG(ATG)ATGACCA, *mutdlx6a* sequence ATTGCAAATAATATG(ATG)ATGACCA). Mutated transcripts were synthesized using the SP6 mMessage mMachine kit (Ambion). The quantitative analysis of control, knockdown and rescue experiments are detailed in (S2 Fig) and [18]. Based on experimental results, co-injection of *dlx5a/dlx6a* translation-blocking MOs (0.8 mM each) and resulting severe phenotype specimens were selected for analysis compared to control embryos injected with water.

### Histological analyses

*In situ* hybridization on whole-mount zebrafish embryos were performed as previously described [18].

For whole-mount immunostaining on zebrafish embryos, dechorionated embryos were fixed in 4% paraformaldehyde (PFA) in 1X phosphate buffered saline (PBS) overnight at 4°C, washed in PBST (PBS 0.1% Tween), dehydrated in methanol and stored in methanol 100% at—20°C. The samples were then rehydrated in graded methanol-PBST series and treated with PBDT (PBS 1% DMSO 0.1% Tween). Cells were immunodetected on whole-mount embryos with mouse anti-SV2 monoclonal antibody diluted in PBDT an incubated overnight at 4°C (1/100, AB 2315387, DHBS). After 5 rounds of 30 min washes in PBST, embryos were incubated overnight at 4°C with secondary anti-mouse HRP-conjugated antibody diluted in PBST (1:200, Jackson Immuno), washed 5 times 30 min in PBST and revealed with DAB chromogenic substrate.

For immunostaining on cryosections, mouse foetuses were fixed 3h in 4% PFA 0.5% Triton X-100 at 4°C, washed overnight at 4°C in PBST, cryopreserved in 30% sucrose in PBS and embedded in OCT for 12–16 μm sectioning with a Leica cryostat. Cryosections were dried for 30 min and washed in PBS. Rehydrated sections were blocked for 1h in 10% normal goat serum, 3% BSA, 0.5% Triton X-100 in PBS. Primary antibodies were diluted in blocking solution and incubated overnight at 4°C (mouse monoclonal Tnnt3 antibody, 1/200, T6277, Sigma; mouse monoclonal Tuj1 antibody, 1/1000, BLE801202, Ozyme). After 3 rounds of 15 min washes in PBST, secondary antibodies were incubated in blocking solution 2h at RT together with 1μg/ml Hoechst 33342 to visualize nuclei. Secondary antibodies consisted of Alexa 488 or 555 goat anti-mouse isotype specific (1/500, Jackson Immunoresearch). After 3 rounds of 15 min washes in PBST, slides were mounted in 70% glycerol for analysis.

Skeletal preparations on mouse foetuses were performed as previously described [34].

## Results

### *Dlx5/6* inactivation induces posterior axis malformations in zebrafish and mouse

**Fig 1:**
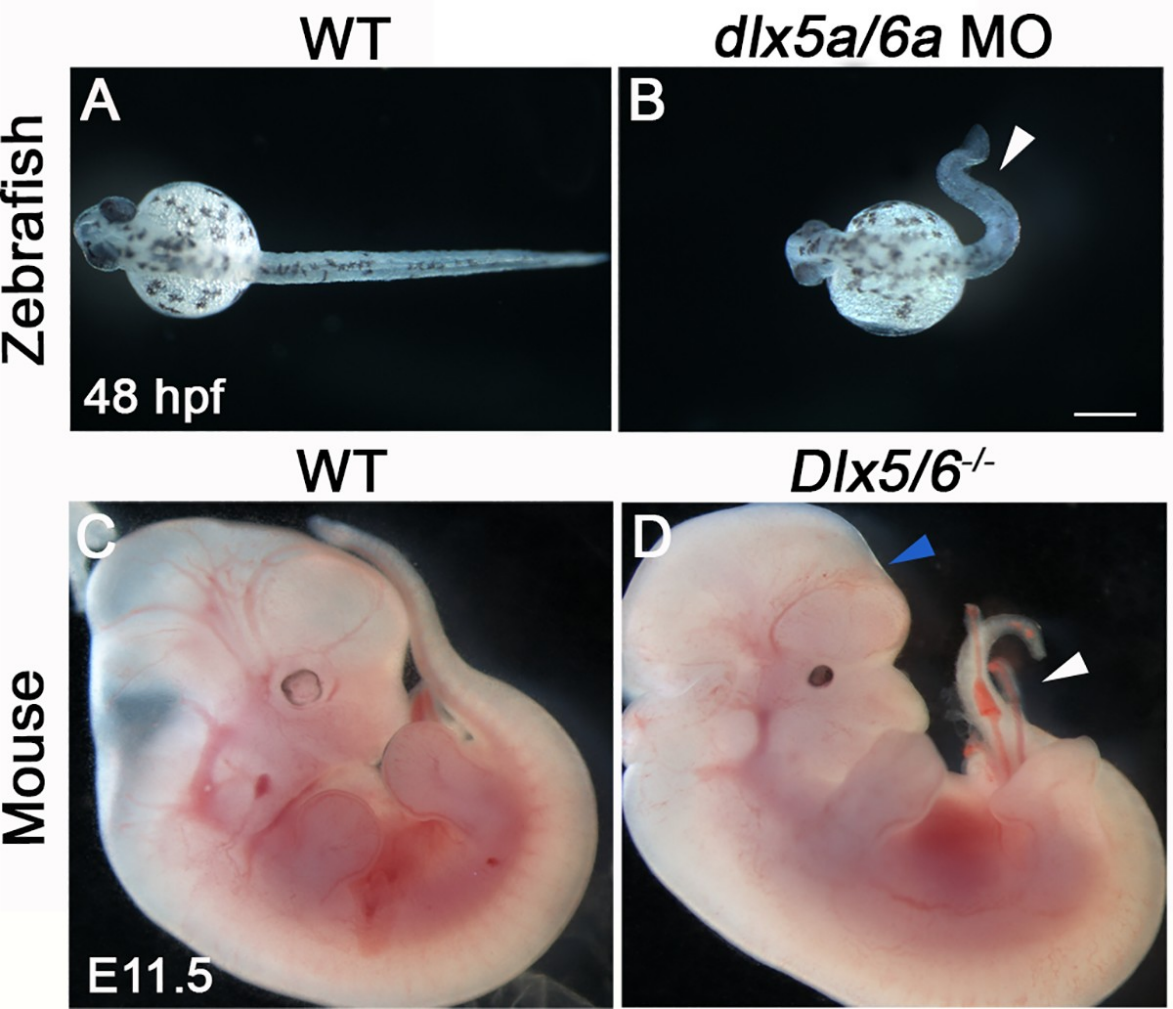
Early phenotype of *Dlx5/6*-inactivated zebrafish and mouse. (A-B) Phenotype of wild type (WT) and *dlx5a/6a* morphant zebrafish at 48 hpf (n>750 for each condition). (C-D) Phenotype of WT and *Dlx5/6*^*-/-*^ mutant mice at E11.5 (n = 6 for each condition). Inactivation of *Dlx5/6* in zebrafish and mouse leads to early defect of posterior axis development characterized by curly-shaped tail phenotype in both models (B, D, white arrowheads). In *Dlx5/6*^*-/-*^ embryos, the caudal phenotype is associated with defect of brain formation (D, blue arrowhead). Scale bar in B for A-B 100 μm, for C-D 1000 μm.

**Fig 2:**
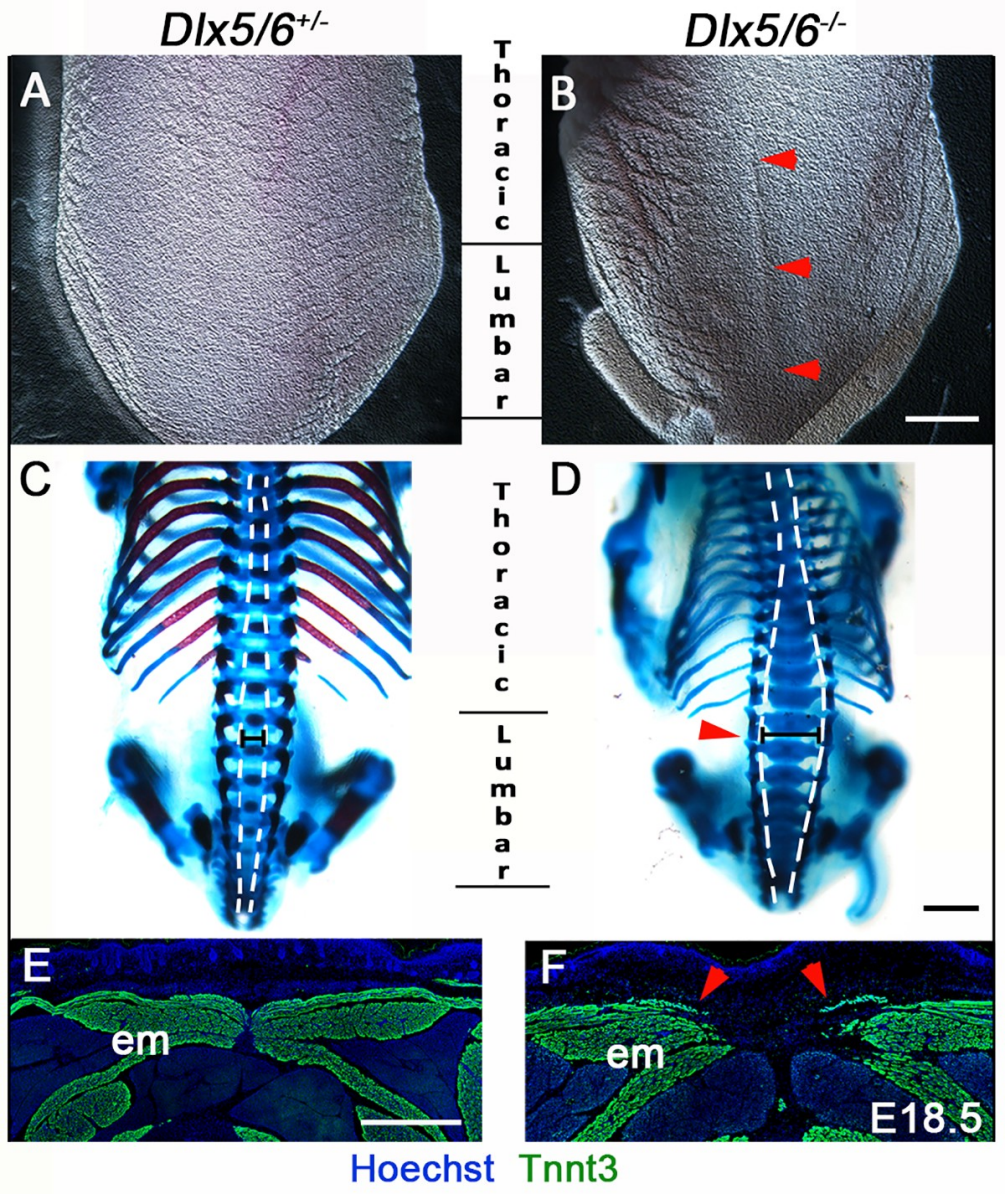
Dorsal midline defects in perinatal *Dlx5/6*^*-/-*^ mice. (A-D) Macroscopic dorsal view (A-B) and skeletal preparation (C-D) of the posterior axis of control *Dlx5/6*^*+/-*^ and *Dlx5/6*^*-/-*^ mutant mice at E18.5 (n = 6 for each condition). (E-F) Immunostaining on coronal cryosections for Tnnt3 in dorsal musculature of control *Dlx5/6*^*+/-*^ and *Dlx5/6*^*-/-*^ E18.5 foetuses (n = 3 for each condition). *Dlx5/6*^*-/-*^ mutants display a dorsal split already evident at macroscopic inspection (B, red arrowheads). This phenotype is associated with defects of thoracic/lumbar vertebrae (D, red arrowhead) and of epaxial muscle formation at the dorsal midline (F, red arrowheads). Abbreviations: em, epaxial muscles. Scale bars in B and D 2000 μm and in E 200 μm.

Axial phenotypes were analysed in *dlx5a/6a* zebrafish morphants and *Dlx5/6*^-/-^ mouse embryos; a curly-shaped tail phenotype was observed in both species (Fig 1A–1D, white arrowheads). 80% mutant mouse embryos presented a curly tail associated with varying degrees of exencephaly (Fig 1, blue arrowhead); the latter phenotype is known to result from defective anterior neural tube closure [17, 19, 25, 35]. The curly tail phenotype was invariably associated to a medio-dorsal split in the thoracic/lumbar region (Fig 2A and 2B, red arrowheads). Skeletal preparations and immunostainings revealed open vertebral arches dorsally at both thoracic and lumbar levels, characteristic of NTDs, and failure of epaxial muscle formation at the dorsal midline (Fig 2C–2F, red arrowheads).

### Expression of *Dlx5* during zebrafish and mouse posterior axis formation

**Fig 3:**
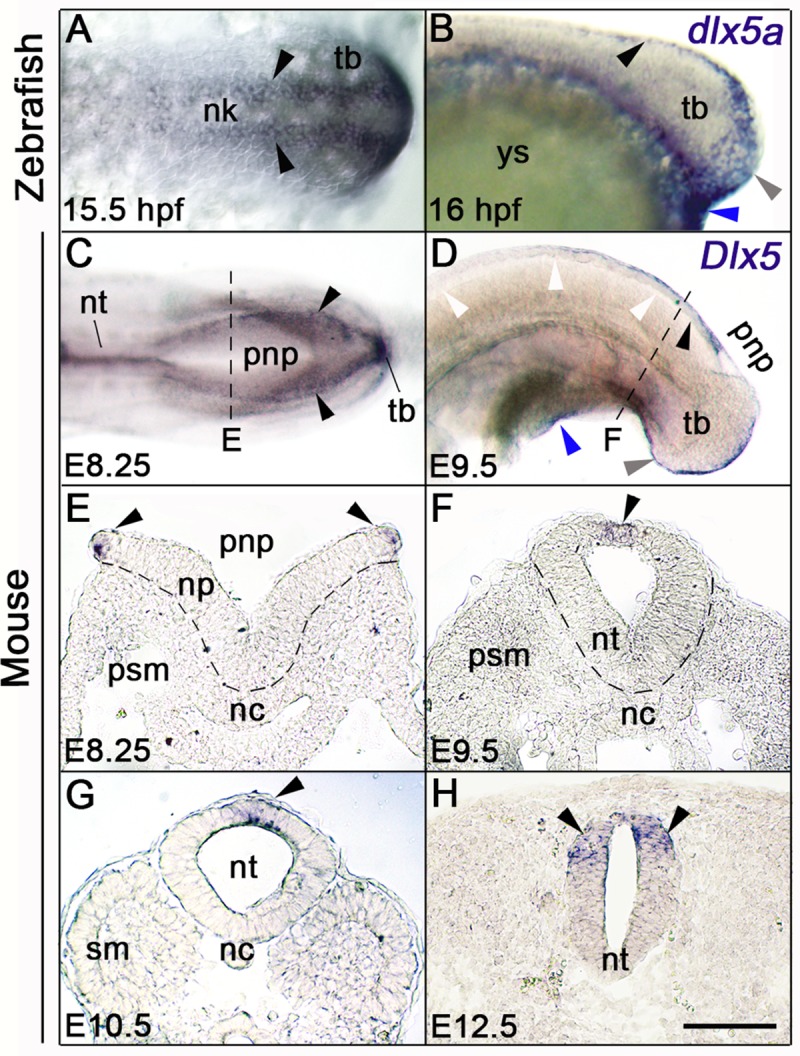
*Dlx5* expression analysis during zebrafish and mouse posterior neurulation. (A-D) Whole-mount *in situ* hybridization for *dlx5a* and *Dlx5*; (A, C) dorsal and (B, D) lateral views of the posterior axis of 15.5 hpf and 16 hpf zebrafish (A-B) and E8.25 and E9.5 mouse embryos (C-D). (E-H) *In situ* hybridization for *Dlx5* on coronal cryosections at the levels indicated by the dashed lines in (C, D and S1 Fig). In zebrafish embryos, *dlx5a* transcripts are detected in NPB cells along neural keel at 15.5 hpf and at the dorsal midline at 16 hpf (A-B, black arrowheads). During mouse posterior neurulation, *Dlx5* is expressed in NPB cells surrounding the posterior neuropore and along the dorsal midline of the neural tube after neural tube closure (C-H, black arrowheads). In both species, *Dlx5* is also detected in the ventral ectodermal ridge of the tail bud and at the cloacal level (grey and blue arrowheads respectively in B, D) (n>10 for each conditions). Abbreviations: nc, notochord; nk, neural keel; np, neural plate; nt, neural tube; pnp, posterior neuropore; psm, presomitic mesoderm; sm, somitic mesoderm; tb, tail bud; ys, yolk sac. Scale bar in H for A, H 50 μm, for B, E-G 75 μm, for C 150 μm, for D 200 μm.

To understand the origin of the axis phenotype observed in *Dlx5/6*-inactivated specimens, we then compared the spatio-temporal expression of *Dlx5* during zebrafish and mouse posterior neurulation. We previously showed in zebrafish that *dlx5a*-expressing ectodermal cells are laterally connected to the neural ectoderm to form the presumptive median fin fold [18]. At 15.5 hpf, *dlx5a*-expressing NPB cells along the neural keel follow a medial convergence toward the dorsal midline during neural rod formation at 16 hpf (Fig 3A and 3B, black arrowheads). At later stages, *dlx5a* expression is limited to median fin fold ectodermal cells at 24 hpf and 48 hpf, and gradually decreased until 72 hpf [18].

Similarly, in mouse embryos, *Dlx5* transcripts were detected in NPB cells surrounding the posterior neuropore and at the dorsal midline after neural tube closure at E8.25 and E9.5 (Fig 3C–3F, black arrowheads), with gradual decrease of expression in a rostro-caudal manner (Fig 3D, white arrowheads). At E10.5 and E12.5, *Dlx5* expression was maintained in the dorsal neural tube after posterior neuropore closure (Fig 3G and 3H, black arrowheads; S1 Fig). In both models, we also observed *Dlx5* expression in the ventral ectodermal ridge (VER) of the tail bud and at the cloacal level (Fig 3B and 3D; S1 Fig, grey and blue arrowheads respectively).

### Posterior neurulation defects in *dlx5a/6a* zebrafish morphants

To further investigate the role of *Dlx5/6* during posterior neurulation, we next performed molecular analyses in early *dlx5a/6a* zebrafish morphants during neural keel-rod transition at 16 hpf, shortly after the onset of *dlx5a* expression at the neural plate border (Fig 3B) [18]. Using translation-blocking MOs against *dlx5a* and *dlx6a*, we obtained a high proportion (74%) of severe curly tail phenotypes compared to control and rescued embryos (S2 Fig) [18]. Given the key role for cell adhesion molecules (CAMs) in neural tube morphogenesis, neural tube closure and epithelial-to-mesenchymal transition [10, 36–39], we analysed the expression of *ncad* (*cdh2*) and *ncam3*, members of the CAM family involved in cell-cell adhesion. We also analysed expression of *msx1b*, marker of NPB cells and premigratory neural crest cells (NCCs) [40, 41], and expression of *foxd3*, marker of premigratory and early migratory NCCs [42].

**Fig 4:**
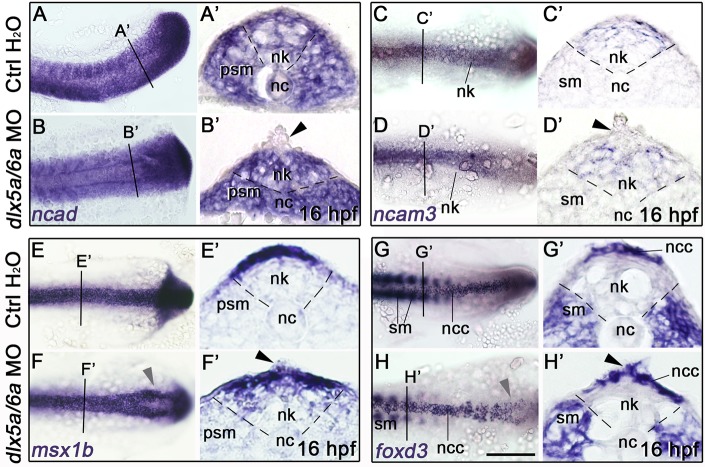
Altered posterior neurulation in early *dlx5a/6a* zebrafish morphants. (A-H) Dorsal views of whole-mount *in situ* hybridization for *ncad*, *ncam3*, *msx1b* and *foxd3* in controls (injected with water) and *dlx5a/6a* morphants at 16 hpf (n>8 for each condition). (A’-H’) *In situ* hybridization for *ncad*, *ncam3*, *msx1b* and *foxd3* on coronal cryosections of 16 hpf controls and *dlx5a/6a* morphants at levels indicated by lines in (A, H). During neural keel-rod transition, the *dlx5a/6a* morphants show a decrease or loss of *ncad*, *ncam3* and *msx1b* in aberrant protruding *foxd3*-positive NCC at the dorsal midline of the neural keel (black arrowhead in B’-H’). The morphants also present a delay in neural keel-rod transition characterized by bifid stripes of expression caudally (F-H, grey arrowheads). Abbreviations: nc, notochord; ncc, neural crest cells; nk, neural keel; psm, presomitic mesoderm; sm; somitic mesoderm. Scale bar in H for A-H 100 μm, for A’-H’ 25 μm.

In 16 hpf control embryos, *ncad* is constitutively expressed in the neural keel and the presomitic/somitic mesoderm. In contrast, *ncam*3 expression is limited to the dorsal part of the neural keel (Fig 4A, 4A’, 4C and 4C’). In *dlx5a/6a* morphants, we observed a loss of *ncad* and *ncam*3 expression in aberrant protruding cells at the dorsal midline of the neural keel (Fig 4B’ and 4D’, black arrowheads). Moreover, whole-mount expression patterns of *msx1b* and *foxd3* in morphants revealed a defect of neural tube formation, with bifid stripes of expression in the caudal-most part of the axis, characteristic of a delay in neural keel-rod transition (Fig 4E–4H, grey arrowheads). On sections, *msx1b* showed a decrease of expression at the midline where protruding cells were detected in morphants (Fig 4E’–4F’, black arrowhead). At 16 hpf, *foxd3* expression in the dorsal neural tube labelled premigratory NCCs (Fig 4G’). The analysis of *dlx5a/6a* morphants revealed that the aberrant protruding cells at the dorsal midline of the neural keel were positive for *foxd3* expression (Fig 4H’, black arrowhead). The results indicated that disrupted *dlx5a/6a* function affects *msx1b* and CAMs (*ncad/ncam3*) expression at the roof plate of the neural keel in aberrant delaminating *foxd3*-positive NCCs.

**Fig 5:**
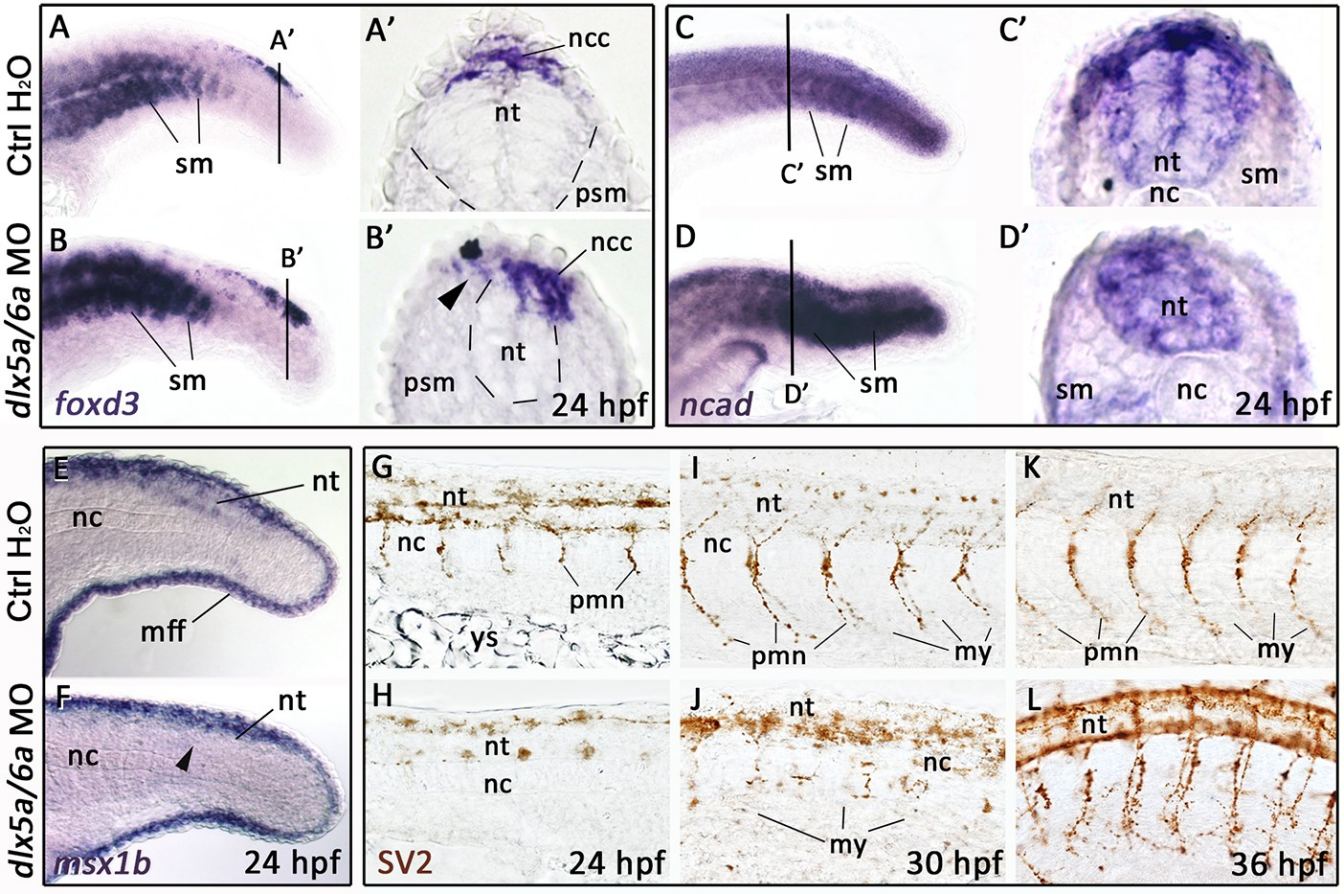
Defects of neural tube and motoneuron formation in *dlx5a/6a* zebrafish morphants. (A-F) Lateral views of whole-mount *in situ* hybridization for *foxd3*, *ncad* and *msx1b* in controls and *dlx5a/6a* zebrafish morphants at 24 hpf (n>8 for each condition). (A’-D’) *In situ* hybridization for *foxd3* and *ncad* on coronal cryosections at levels indicated by lines in (A-D). The *dlx5a/6a* morphants show a reduced neural tube (A’-D’) associated with a defect of NCC migration (B’, black arrowhead), aberrant accumulation of *foxd3* and *ncad* transcripts in somites (B, D) and decrease of *msx1b* in the neural tube (F, black arrowhead). (G-L) Lateral views of whole-mount immunostaining for SV2 in 24 hpf, 30 hpf and 36 hpf control and *dlx5a/6a* morphant embryos (n>8 for each condition). In *dlx5a/6a* morphants at 24 hpf and 30 hpf, axonal projections of primary motoneurons fail to form (H-J). In 36 hpf morphants, primary motoneurons show abnormal synaptic connections to their myotomal targets (L). Abbreviations: mff, median fin fold; my, myotomes; nc, notochord; ncc, neural crest cells; nt, neural tube; pmn, primary motoneurons; psm, presomitic mesoderm; sm; somitic mesoderm, ys, yolk sac. Scale bar in A for A-F 100 μm, for A’-D’ 25 μm and for G-L 50 μm.

We then studied the effect of *dlx5a/6a* inactivation at later stages of neural tube formation. In 24 hpf controls, dorsal *foxd3* expression in migratory NCCs was observed in the caudal neural tube (Fig 5A and 5A’). In *dlx5a/6a* morphants, *foxd3*-positive cells showed abnormal asymmetric profile (Fig 5B’, black arrowhead) associated with a reduced neural tube and defect of lumen formation (Fig 5A’–5B’, dashed lines), the latter phenotype being well revealed by the constitutive *ncad* expression in the neural tissue (Fig 5C’–5D’). Beside NCCs, *foxd3* and *ncad* transcripts were also detected in somites (Fig 5A and 5C); their expression was strongly increased in *dlx5a/6a* morphants. In control embryos, somites were arranged as V-shaped chevrons, while in *dlx5a/6a* morphants the somite boundaries presented abnormal U-shaped chevrons (Fig 5A–5D, dashed lines). A similar defect of somitic segmentation was also observed at earlier stages of development thanks to the analysis of *fgf8a* expression, a marker of anterior somitic mesoderm (S3 Fig). The expression profile of *msx1b* along the neural tube was also altered in *dlx5a/6a* MO embryos at 24 hpf (Fig 5E and 5F, black arrowhead), while expression in the median fin fold compartment seemed unaffected.

We next analysed the primary motoneuron (PMN) population in *dlx5a/6a* morphants; these cells originate from NCCs and are known to be affected in NTDs [43, 44]. In 24 hpf controls, PMNs are characterized by synaptic vesicle SV2 expression; axonal projections start to elongate from the neural tube to reach their myotomal targets, forming the neuromuscular junctions at 30 hpf and 36 hpf (Fig 5G, 5I and 5K). In contrast, *dlx5a/6a* morphants showed defect of motoneuronal outgrowth at 24 hpf and PMNs failed to connect the myotomes at 30 hpf (Fig 5H and 5J). At 36 hpf, axonal projections were completed but PMNs showed defective neuromuscular junctions with aberrant synaptic arborescence (Fig 5L). PMN defects were associated with SV2 protein accumulation in the dorsal neural tube at 30 hpf and 36 hpf (Fig 5J and 5L). Abnormal neuromuscular innervation was also present in *Dlx5/6* mutant mice as shown by immunostainings for the neuronal marker Tuj1 in trunk epaxial muscles positive for Tnnt3 (S4 Fig, white arrowheads).

We also studied the expression of *shha* and *bmp4* that are organizers of tail development [11, 45–48]. In 24 hpf controls, *shha* is expressed in the notochord, the neural tube floor plate and in the tail stem cell pool, namely the chordoneural hinge (S3 Fig). At 48 hpf, *shha* expression is limited to the floor plate (S3 Fig). In morphants, *shha* expression was maintained but well reveals the undulating phenotype of axial structures (S3 Fig). While *bmp4* did not show obvious defects of expression at 16 hpf and 24 hpf [18], expression in the spinal cord was altered at 48 hpf (S3 Fig).

Altogether, our data indicated that disrupted *dlx5a/6a* function in zebrafish led to a loss of CAM expression in protruding NCCs at the dorsal midline, resulting in posterior neural tube defects and mispatterned NCC-derived primary motoneurons.

## Discussion

*Dlx5* is one of the earliest NPB markers during gastrulation [20, 21, 24]. Particular attention has been paid to its role in anterior neural tube formation and in the specification of the border between non-neural and neural plate territories [11, 21, 35, 49, 50]. However, the implication of *Dlx5/6* during posterior neurulation has never been reported.

Our analysis highlights the role of *Dlx5/6* in NPB cells during posterior neurulation in zebrafish and mouse (Figs 1–5) [18]. In both models, *Dlx6* expression was undetectable by *in situ* hybridization during early neurulation. In zebrafish, *dlx6a* expression is observed in the presumptive median fin fold that is related to *dlx5a*-expressing NPB cells during unpaired fin development [18]. *Dlx6* expression appears delayed and weaker compared to expression of the *Dlx5* paralog, a difference that was observed by others and us in vertebrate embryos using a variety of probes for these genes. The inactivation of both genes is however required to observe a fully penetrant phenotype, underlying the redundant functions of *Dlx* paralogs in vertebrates (S2 Fig) [16–19, 51–53].

Expression analyses for *Dlx* homologs in various models including chick, xenopus, lamprey and amphioxus suggest that *Dlx* expression in NPB cells has been established early during chordate evolution [21, 24, 49, 54, 55]. However, we can still notice differences in *Dlx5* expression along the rostro-caudal axis of zebrafish and mouse. In mouse, *Dlx5* is expressed in NPB cells during both anterior and posterior neurulation (Fig 3) [20]. In amphioxus, *amphiDll* is expressed in NPB cells all along the axis, suggesting a conserved role of *Distal-less-*related genes in anterior and posterior neurulation in chordates [55]. In contrast, in zebrafish, the anterior limit of *dlx5a*-expressing NPB cells is located at the 8^th^ somite level and appears closely related to the establishment of the presumptive median fin fold (Fig 3) [18]. According to our expression analysis, we did not find evidence of anterior neurulation defects in *dlx5a/6a* morphants [29]. In teleosts, neurulation is characterized by uniform epithelial condensation and cavitation, which give rise to both anterior and posterior neural tube [6, 8]. It has been suggested that zebrafish might present primitive mechanisms of neurulation, however basal chordates show primary and secondary neurulation as observed in higher vertebrates [6, 56]. Our results indicate that, while the cellular morphogenetic basis of neural tube formation is uniform along the rostro-caudal axis of zebrafish, the anterior and posterior neurulation processes do not involve same molecular mechanisms, suggesting evolutionary divergence of *Dlx5/6* function during anterior neural tube formation in teleosts. Our data bring new insights into the genetic and evolutionary origins of neural tube formation in chordates. Special attention should be paid in future studies to elucidate the genetic requirement differences between anterior and posterior neurulation in teleosts.

Our analysis reveals that *Dlx5/6* inactivation in zebrafish and mouse leads to early curly tail phenotypes in both models (Fig 1). In *Dlx5/6*^-/-^ mice, the tail phenotype is associated with midline axis defects and brain malformations characteristic of NTDs (Figs 1 and 2) [17, 19, 25]. Intriguingly, the axis defects observed in *Dlx5/6*^*-/-*^ mice was similar to the phenotype of *CT “curly tail”* mutants, a historical model of NTDs [5, 57, 58]. The data thus suggested that the axis phenotype observed in *Dlx5/6*-inactivated zebrafish and mice resulted from defects of posterior neurulation, an aspect that we confirm through our functional analysis in zebrafish.

We demonstrate that *dlx5a/6a* morphants present neural tube defects associated with aberrant dorsal delamination and migration of NCCs (Figs 4 and 5). The protruding NCCs observed at the dorsal midline of the neural keel show loss of CAM expression (Fig 4). Cell adhesion molecules are important actors during neurulation [7]. In particular, *ncad* is required for NPB convergence, neural tube closure, maintenance of neural tube integrity and epithelial-to-mesenchymal transition [10, 36–39]. In zebrafish, *ncad* inactivation represses neural tube formation due to defect of convergence and intercalation of NCCs [10, 36, 37]. Our data thus indicate that *dlx5a/6a* genes act in NPB cells for adhesion integrity of NCCs during neural tube formation.

It has been previously demonstrated that *Dlx5* specifies NPB cells during cranial neural tube formation in mouse and chick [20, 21, 24]. However, *Dlx* activity in xenopus is not necessary for induction of NCCs [49]. Our findings confirm that *Dlx* inactivation does not impact on NCC induction as *foxd3* expression is maintained in the protruding NCCs observed in *dlx5a/6a* morphants (Fig 4). In addition, *msxb1* expression is decreased in the protruding NCCs of *dlx5a/6a* morphants, associated with defects in primary motoneurons development (Figs 4 and 5). It has been shown that *msx* and *ncad* genes are required during zebrafish neurogenesis [37, 40]. The neuromuscular deficiencies observed in *dlx5a/6a* morphants may result from altered expression of *msx1b* and CAMs in premigratory NCCs. In mouse, *Dlx5* and *Msx2* genes regulate anterior neural tube closure through expression of EphrinA5-EphA7 involved in cell adhesion [35]. This suggests that a common genetic network implicating *Dlx*, *Msx* and cell adhesion molecules is involved in neural plate border and neural crest cells during mouse and zebrafish neurulation.

Taken together, our findings reveal that *Dlx5/6* genes are required during vertebrate posterior axis formation. The results show that *Dlx5* expression is limited to NPB and VER cells (including the cloacal derivative) during posterior neurulation. The VER is known to act as a signalling centre during tail somitogenesis and elongation [59, 60]. VER cells undergo epithelial-to-mesenchymal transition during tail development as observed dorsally in NCCs [61]. The continuous dorso-ventral ectodermal *Dlx5*-positive domain might reflect that the VER represents a ventral extension of dorsal ectodermal cells during tail morphogenesis. The inhibition of BMP signalling by *Noggin* suppresses the VER epithelial-to-mesenchymal transition process during tail morphogenesis [59, 61]. In *dlx5a/6a* morphants, ectodermal *Bmp4* expression is not affected during early posterior axis development [18], and the expression of *Noggin* has been shown to be independent of *Dlx5* during craniofacial development [62]. The data thus indicate that BMP4 signalling is regulated independently of *Dlx5* during early posterior axis formation. Moreover, *Dlx5/6* expression in the VER does not appear required for tail elongation as both *Dlx5/6*^-/-^ mice and *dlx5a/6a* morphants show equivalent number of axial segments compare to controls. However, as zebrafish morphants present abnormal somite boundaries associated to overexpression of *foxd3* and *ncad* in posterior somites (S3 Fig, Fig 5), *Dlx5/6* expression in the VER might ensure proper regulation of somitogenesis.

These new results give insights for a better understanding of the cellular and molecular processes that could be altered in some human congenital NTDs, such as craniorachischisis that originates from defects of both anterior and posterior neurulations. The anterior and posterior NTDs observed in *Dlx5/6* mutant mice are also associated with hypospadias, characterized by midline urethral malformations, and limb ectrodactyly [17, 31, 32]. Expression of *Dlx5/6* in the cloacal membrane, linked to the ventral ectodermal ridge [61], and in the genital tubercle is necessary for urethral formation. Moreover, it has been shown that *Dlx5/6* expression in the apical ectodermal ridge is required for proper appendage formation in mouse and zebrafish [17, 18, 32]. The limb phenotype of *Dlx5/6*^*-/-*^ mice resembles that of patients with congenital split hand-foot malformation type I (SHFM-I), linked to genomic deletion or rearrangement in the *DLX5/DLX6* cluster locus. Altogether, the data unveil the role of *Dlx5/6* in ectodermal cells for the proper development of the neural tube, the urogenital system and limbs. It has been reported that NTDs can be associated with limb malformations and other midline defects, including urogenital and diaphragmatic disorders, as observed in Czeizel-Losonci syndrome [63–67]. Our data bring new light on common etiology for a spectrum of idiopathic anomalies characterizing certain human congenital disorders.

## Supporting information

S1 Fig *Dlx5* expression after posterior neuropore closure in mouse. (A, B) Lateral views of whole-mount *in situ* hybridization for *Dlx5* in E10.5 and E12.5 mice. The dashed lines indicate the section levels analysed in Fig 3G and 3H. *Dlx5* expression is detected at the cloacal level and in the VER at E10.5 (A, blue and grey arrowheads respectively) but is not detectable at E12.5. Abbreviations: nt, neural tube; sm, somitic mesoderm; tb, tail bud; ver, ventral ectodermal ridge. Scale bar in A for A-B 200 μm.
(TIF)

S2 Fig Phenotypes resulting from the morpholino knockdown and mRNA rescue experiments. Proportion of normal (blue), moderate (purple) and severe (red) phenotypes observed at 48 hpf after morpholino knockdown and mRNA rescue experiments. The number (n) of specimens analysed is indicated for each treatment. Treatments: control embryos injected with H_2_O; control embryos injected with a control standard MO (1.6 mM); single morphants injected with either *dlx5a* or *dlx6a* translation-blocking MOs (0.8 mM); double morphants co-injected with *dlx5a* and *dlx6a* e2i2 splice-blocking MOs (0.4 mM each); double morphants co-injected with *dlx5a* and *dlx6a* translation-blocking MOs at two different concentrations (0.4 mM or 0.8 mM each); control embryos injected with *GFP* mRNA (200 ng/μl); embryos co-injected with *dlx5a/6a* translation-blocking MOs (0.8 mM each) and *GFP* mRNA (200 ng/μl) and embryos co-injected with *dlx5a/6a* translation-blocking MOs (0.8 mM each) and mutated *dlx5a/dlx6a* mRNAs (70 ng/μl each). Scale bar 100 μm.
(TIF)

S3 Fig Expression of *fgf8a, shha* and *bmp4* during zebrafish posterior neurulation. (A-H) Lateral views of whole-mount *in situ* hybridization in controls (injected with water) and *dlx5a/6a* zebrafish morphants for *fgf8a* at 16 hpf (A-B), *shha* at 24 hpf and 48 hpf (C-F) and for *bmp4* at 48 hpf (G-H) (n>8 for each condition). The inactivation of *dlx5a/6a* leads to defects of somite boundaries as highlighted by *fgf8a* expression in the anterior somitic mesoderm at 16 hpf (A-B). Expression of *shha* in the notochord and neural tube floor plate well reveals the ondulating axis phenotype observed in *dlx5a/6a* morphants (C-F). At 48 hpf, the axis malformation is associated with a decrease of *bmp4* expression in the spinal cord (G-H). Abbreviations: cn, chordoneural hinge; fp, floor plate; mff, median fin fold; my, myotomes; nc, notochord; nt, neural tube; sm, somites; sp, spinal cord. Scale bar in G for A-B 75 μm, for C-H 100 μm.
(TIF)

S4 Fig Defect of neuromuscular innervation in *Dlx5/6^-/-^* mice. (A-B) Immunostaining on coronal cryosections for Tnnt3 and Tuj1 in epaxial muscles of E18.5 control and *Dlx5/6*^*-/-*^ foetuses (n = 3 for each condition). The *Dlx5/6*^*-/-*^ mutants show defect of neuromuscular innervation in epaxial musculature. Abbreviations: epm, epaxial muscles. Scale bar in A for A-B 20 μm.
(TIF)
