# Supplementary figures and images for: Posterior axis formation requires Dlx5/Dlx6 expression at the neural plate border
Source: PLoS One. 2019 Mar 19;14(3):e0214063. doi: 10.1371/journal.pone.0214063 (PMC6424422; doi:10.1371/journal.pone.0214063)

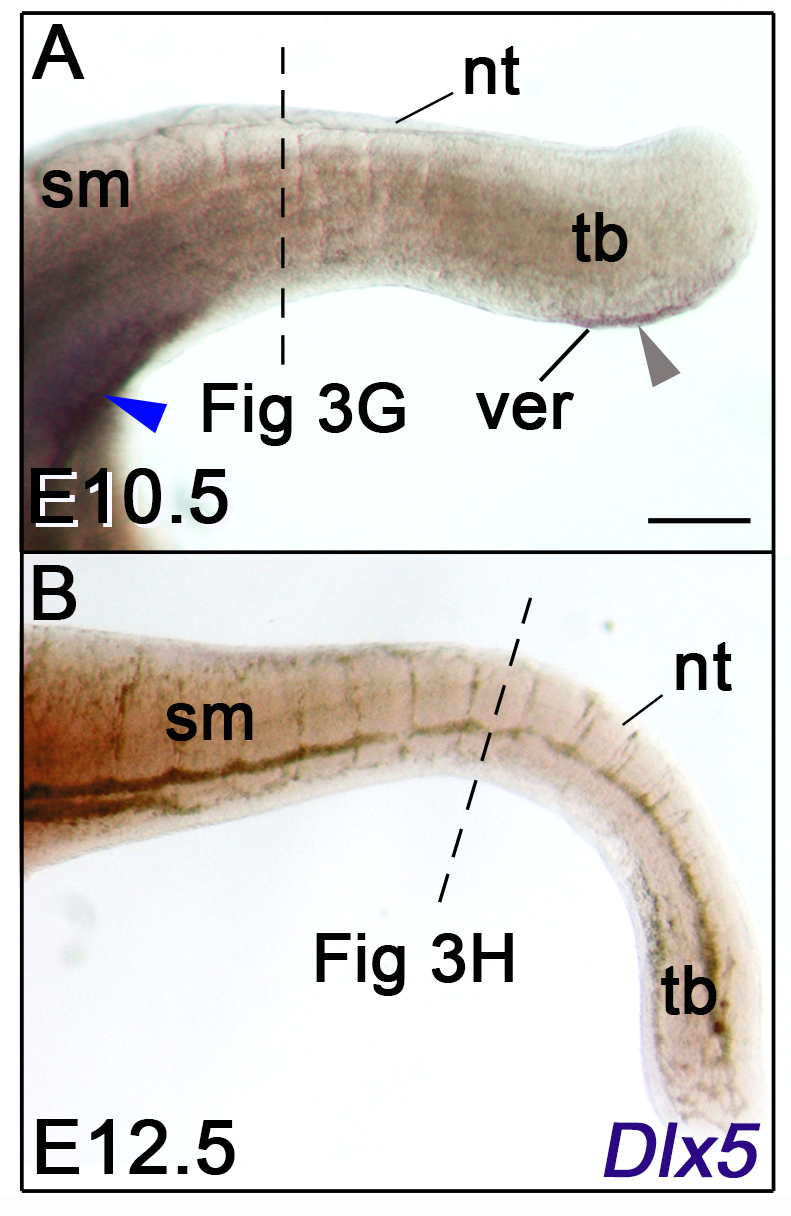

Supplement: S1 Fig — (A, B) Lateral views of whole-mount in situ hybridization for Dlx5 in E10.5 and E12.5 mice. The dashed lines indicate the section levels analysed in Fig 3G and 3H. Dlx5 expression is detected at the cloacal level and in the VER at E10.5 (A, blue and grey arrowheads respectively) but is not detectable at E12.5. Abbreviations: nt, neural tube; sm, somitic mesoderm; tb, tail bud; ver, ventral ectodermal ridge. Scale bar in A for A-B 200 μm. (TIF) [file pone.0214063.s001.tif]

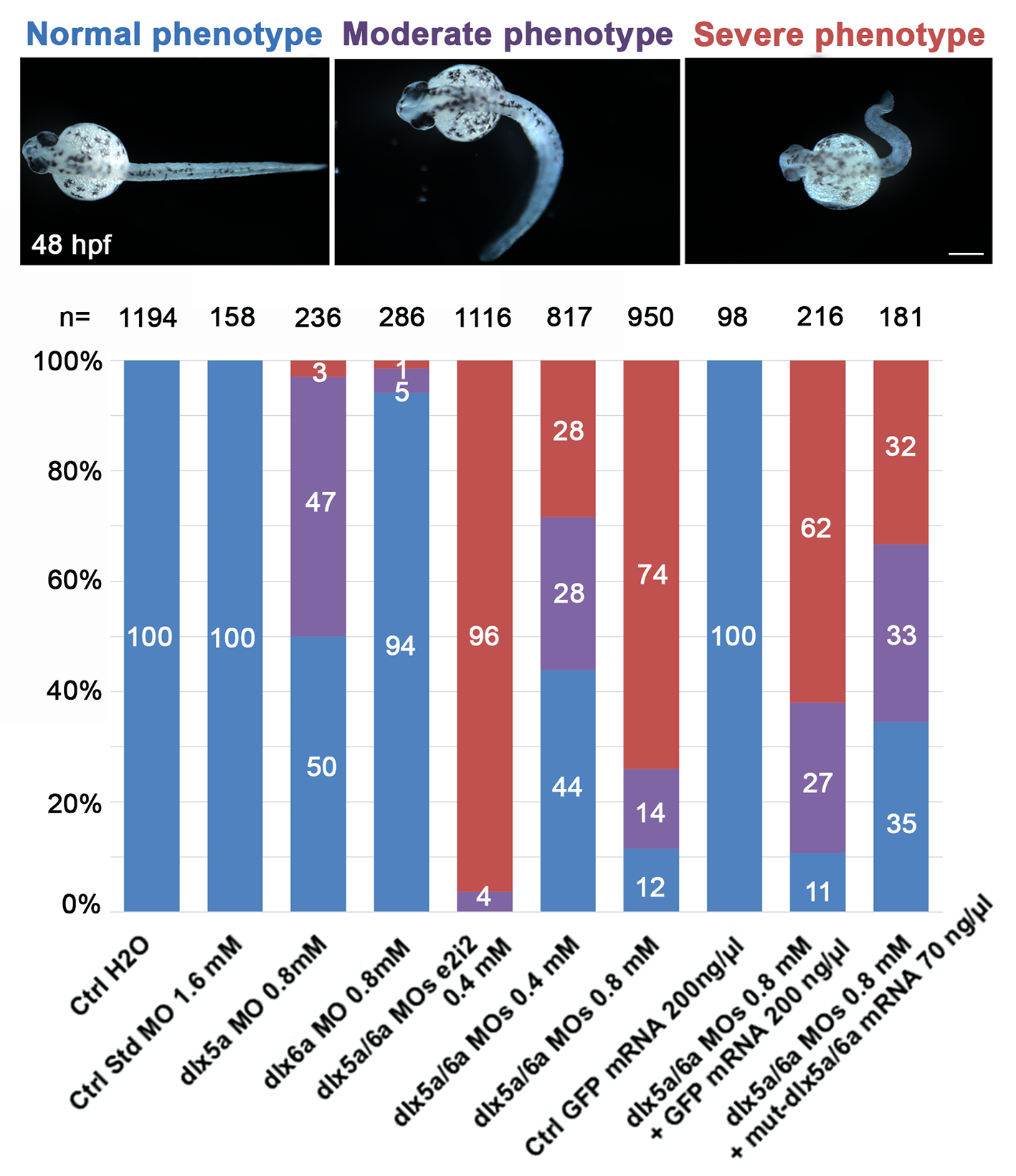

Supplement: S2 Fig — Proportion of normal (blue), moderate (purple) and severe (red) phenotypes observed at 48 hpf after morpholino knockdown and mRNA rescue experiments. The number (n) of specimens analysed is indicated for each treatment. Treatments: control embryos injected with H2O; control embryos injected with a control standard MO (1.6 mM); single morphants injected with either dlx5a or dlx6a translation-blocking MOs (0.8 mM); double morphants co-injected with dlx5a and dlx6a e2i2 splice-blocking MOs (0.4 mM each); double morphants co-injected with dlx5a and dlx6a translation-blocking MOs at two different concentrations (0.4 mM or 0.8 mM each); control embryos injected with GFP mRNA (200 ng/μl); embryos co-injected with dlx5a/6a translation-blocking MOs (0.8 mM each) and GFP mRNA (200 ng/μl) and embryos co-injected with dlx5a/6a translation-blocking MOs (0.8 mM each) and mutated dlx5a/dlx6a mRNAs (70 ng/μl each). Scale bar 100 μm. (TIF) [file pone.0214063.s002.tif]

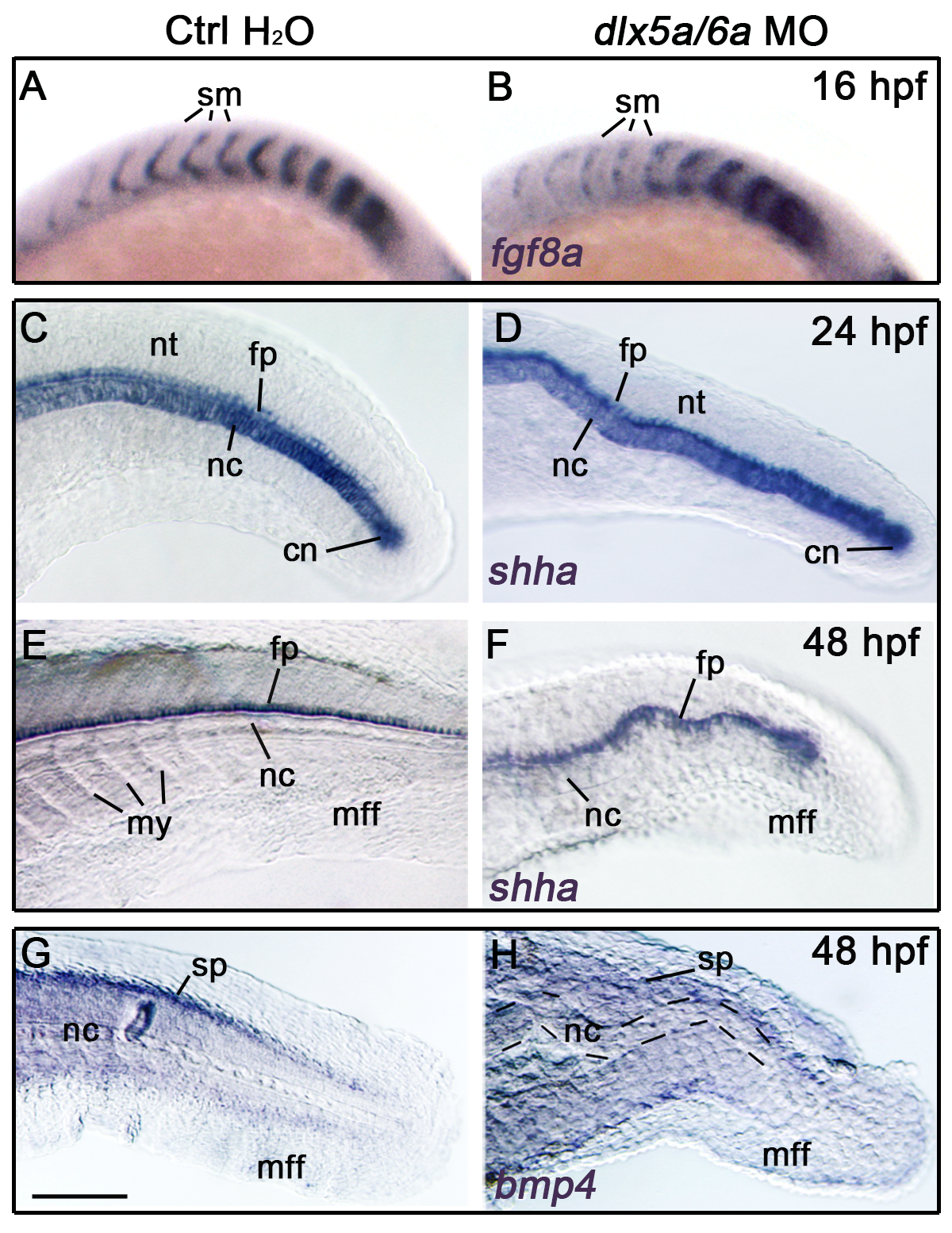

Supplement: S3 Fig — (A-H) Lateral views of whole-mount in situ hybridization in controls (injected with water) and dlx5a/6a zebrafish morphants for fgf8a at 16 hpf (A-B), shha at 24 hpf and 48 hpf (C-F) and for bmp4 at 48 hpf (G-H) (n>8 for each condition). The inactivation of dlx5a/6a leads to defects of somite boundaries as highlighted by fgf8a expression in the anterior somitic mesoderm at 16 hpf (A-B). Expression of shha in the notochord and neural tube floor plate well reveals the ondulating axis phenotype observed in dlx5a/6a morphants (C-F). At 48 hpf, the axis malformation is associated with a decrease of bmp4 expression in the spinal cord (G-H). Abbreviations: cn, chordoneural hinge; fp, floor plate; mff, median fin fold; my, myotomes; nc, notochord; nt, neural tube; sm, somites; sp, spinal cord. Scale bar in G for A-B 75 μm, for C-H 100 μm. (TIF) [file pone.0214063.s003.tif]

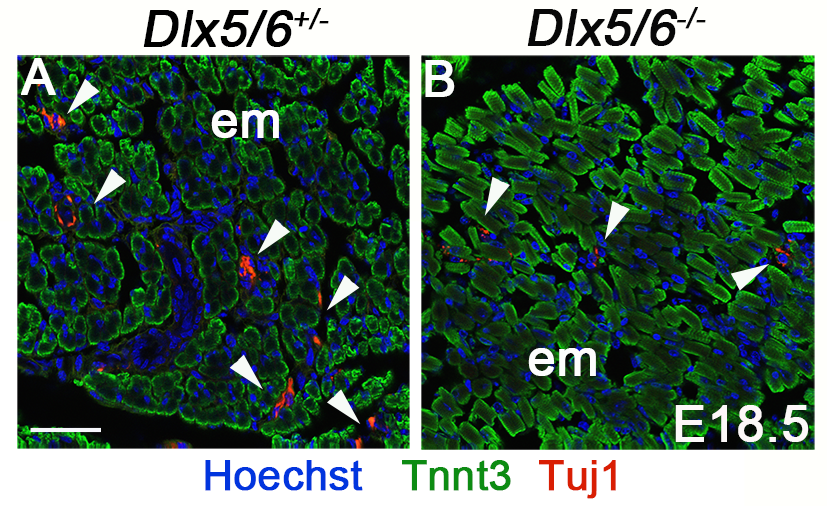

Supplement: S4 Fig — (A-B) Immunostaining on coronal cryosections for Tnnt3 and Tuj1 in epaxial muscles of E18.5 control and Dlx5/6-/- foetuses (n = 3 for each condition). The Dlx5/6-/- mutants show defect of neuromuscular innervation in epaxial musculature. Abbreviations: epm, epaxial muscles. Scale bar in A for A-B 20 μm. (TIF) [file pone.0214063.s004.tif]
